# Individualizing social determinants of health: is educational attainment a community resource?

**DOI:** 10.1007/s10654-025-01332-8

**Published:** 2025-11-21

**Authors:** Whitney Wells, M. Maria Glymour

**Affiliations:** 1 Department of Epidemiology and Biostatistics, University of California, San Francisco, USA; 2 Department of Epidemiology, School of Public Health, Boston University, Talbot Building 326 East, 715 Albany Street, Boston, MA 02118 USA

Walters et al. augment prior observational and quasi-experimental research estimating the effects of education on dementia with novel analyses based on twin comparisons. In this commentary, we first discuss our interpretation of Walters’ results. We then discuss the causal estimands from different study designs, and why twin study estimands may not be of greatest substantive interest.

## Recapping Walters’ empirical results

Walters et al. first contrast mean scores on a “latent dementia index” (LDI) for individuals with different levels of education, using the 9-level ISCED education classifications. When comparing unrelated pairs discordant for education, they estimate a difference of 0.44 to 0.46 LDI units. LDI units are difficult to interpret, but these coefficients correspond to about a third of a standard deviation in LDI. Depending on the correspondence between ISCED units and years of schooling, this coefficient is at the high end of prior estimates of cognitive effects of education among older adults [1]. Inflated estimates are unsurprising given the comparison of unrelated individuals controls for no covariates other than birth year and sex. The authors then estimate LDI contrasts for dizygotic (DZ) and monozygotic (MZ) twins. In these comparisons, effect estimates range from 0.02 to 0.30 LDI units. The sample-size weighted average estimate is 0.14 SD on the LDI scale, roughly in the range of prior estimates of effects of education on cognitive outcomes.

Walters’ presentation highlights the lack of statistical significance, particularly for the contrast of DZ versus MZ twins. This particular significance contrast is not informative however, for several reasons. The MZ sample is small (1568 DZ pairs vs. 880 MZ pairs, with only 79 dementia cases expected among MZ females). LDI has substantial measurement error (sensitivity of 51–87% and specificity of 91–98% for clinical dementia) [2]. Finally, the educational difference for discordant MZ twins was around 35% smaller than the educational difference for discordant DZ twins (Walters Figure S1).

In further analysis, Walters et al. estimate the effect of education based on co-twin control regression models. These models include more twins, but the original ISCED categories were collapsed from nine into only three educational groups, which would lead to fewer discrepant pairs. They report: (1) more highly educated twin pairs have lower dementia risk than less educated twin pairs; (2) lack of statistically significant evidence that twins with more education average lower dementia risk than their co-twins with less education; (3) the advantage associated with more education is larger for DZ than MZ twins. The authors interpret the significant interaction between zygosity and education to indicate that “the familial confounding was largely explained by additive genetic variance in common to the exposure and the outcome.” The grounds for this conclusion are unclear, since the magnitude of the interaction is never compared to the magnitude of ‘familial confounding’.

The key between-twin comparison from Walters’ Table 2, Model 2 (β =−0.07, approximate 95% CI: −0.15, 0.01) suggests modest educational benefits when comparing a twin to their co-twin. This estimate is somewhat smaller than expected based on prior research, and we discuss possible explanations below. Results also reveal the fragility of the estimates from MZ twins (Table 2, Model 3). The point estimate for MZ twins — taken at face value — would indicate that education *increases* dementia risk, but the CI ranges from small benefits to large harms (β = 0.13, approximate 95% CI: −0.05, 0.31).

Walters et al.’s analysis rests on the recognition that multiple pathways — operating via individual or family resources — may connect education and health. Not all study designs identify the same set of causal pathways. We posit that effect estimates from twin designs may be smaller than estimates from other designs in part because twin studies exclude relevant community-level causal paths captured in other study designs.

## Individual versus group social determinants of health

Social determinants of health encompass both individual and group-level exposures. Factors such as social cohesion or neighborhood poverty emerge from group interactions or are defined at a group-level. In contrast, some social determinants, e.g., earnings and occupational prestige, are amenable to measurement at an individual-level and research emphasizes their health benefits for the individual [3, 4].

With respect to education and health, discussions of mechanisms most often invoke individual-level mechanisms, such as increased earnings. Some research also evaluates resources or harms that flow from education of family members, e.g., spouses or adult children [5, 6]. In neighborhood studies, community average education may be used as a proxy for neighborhood disadvantage. Beyond serving as a proxy for disadvantage however, many plausible mechanisms link the education of community members to the health of their neighbors. Highly educated individuals may share knowledge and information, create economic opportunities, establish behavioral norms, and deliver political power to their communities.

Studies estimating the effects of spousal or community education on health, separately from own education, have typically found that coefficients are largest for own-education and smaller but positive for other measured dimensions of education. The magnitude of coefficients varies substantially across health outcomes as well as contextual (e.g. country, cohort) and individual (e.g. gender) characteristics [7–11]. The evidence suggests however that these pathways in combination are important contributors to the overall association of education with health. Yet, literature envisioning educational attainment as a community resource -- with health advantages of educational interventions flowing not just to the individual who completes the schooling, but spilling over to that person’s spouse, children, neighbors, friends, and entire social network -- remains sparse.

### Are educational interventions more like dietary counseling or vaccination campaigns?

The idea that mechanisms for treatment effects might operate through a tangled web of individual- and social-level pathways is not new. Spillover effects and cross-level interactions are well-recognized in vaccines research (e.g., herd immunity) [12, 13]. When evaluating vaccines, disregarding these processes leads to major misinterpretation of individual effect estimates. Modern causal inference frameworks emphasize that to evaluate the effect of an exposure, it is valuable to tie that exposure to a well-defined intervention. We argue that we should not conceptualize the effect of education as we would, say, the effect of eating broccoli. Rather, educational interventions typically trigger pathways via community and family education, including moderation of individual-level processes, and these pathways should be included as part of the causal estimand.

Many, perhaps most, plausible interventions on education are policy changes (opening new educational institutions, increasing admissions rates, increasing financial support available, or increasing school quality): pathways via community or family education are components of the effects of these interventions. Even if the intervention is at the individual-level (e.g., charter school admissions lotteries, individual coaching on college applications), social pathways including spousal selection and moderating effects of community education are still part of the effect.

## What is estimated with different study designs?

Conventional observational study designs contrast the health of individuals with different levels of education. Given correlations between individual, family, and community education, any effect estimate will include some contribution from those processes unless family and community education are comprehensively controlled for (Table 1). The effect estimate will therefore capture effects of education via multiple social pathways, rather than the exclusive direct effect of individual educational attainment on health. The educational intervention this estimate corresponds to may therefore be closer to a policy change with community-level impacts than an individual-level intervention.

**Table 1:** Framework for effect of education, comparison of approaches

Pathway	Conventional covariate control	Compulsory schooling law natural experiments	MZ twin comparison
Direct effect of individual educational attainment on dementia risk	+	+	+
Parent/family factors that influence education and dementia risk	+	-	-
Paths via spousal or children’s education to dementia risk	+	Partial*	+
Effect of average education of community members on dementia risk	+	++	-
Differential effect of individual education, depending on spouse’s or children’s education	+	Partial*	+
Differential effect of individual education, depending on average education of community members	+	++	-

*Schooling laws may differentially influence spouse’s education; in heterosexual marriages men are often a few years older, which could put them in a birth cohort on the opposite side of a schooling law change.

Many studies use changes in state- or country-level mandatory schooling laws to identify causal effects of education on health. Such natural experiments compare individuals born in the same jurisdiction in years before and after a compulsory schooling law increase. Instrument-based approaches like this generally aim to circumvent individual-level confounding such as by family factors, however do not eliminate all social pathways between education and health (Table 1). Schooling laws will also influence spouse’s education. Because schooling laws are a community-level intervention, pathways via community education would likely contribute to the coefficient to a larger extent than the coefficient from a conventional study design. This warrants further reflection on whether instrumental variable estimates should be interpreted as individual effects, and also challenges whether the individual effect estimand should be the target in the first place.

Twin designs such as Walters et al. contrast health outcomes of twins with different individual education. These studies thus control for pathways via the community, but not spouses (Table 1). Twin studies rely on the assumption that one twin’s education does not influence the other’s health outcomes, but as our discussion so far has highlighted, this assumption may not hold [14]. Twin studies also will not capture modifying effects of community average education, because, by definition, it is uncorrelated with individual education of each twin. We would thus expect the twin study estimate to diverge from the estimate using other study designs, and to correspond more closely to an individual-level intervention. Depending on the educational intervention one has in mind, the twin study estimand may not be of greatest substantive interest.

## Take home messages

Researchers often interpret effect estimates from different study designs as estimating the same causal parameter: “the effect” of individual educational attainment on health. Pathways via community or family effects may be presented as biases or ignored. This mismatch between the causal estimands of different study designs may contribute to inconsistent findings. We resist the temptation to endorse a hierarchy of these study designs; each plays an important role in evidence triangulation by addressing particular biases and providing estimates of different but related estimands. For many public health questions, the effects of educational interventions should include pathways due to changes in education at the family and community levels.

## Declarations

### Research involved human and animal participants

This manuscript involved no human subjects.
